# ASSOCIATIONS BETWEEN WALKING SPEED AND GUT MICROBIOME DIVERSITY IN OLDER MEN FROM THE MROS STUDY

**DOI:** 10.1093/geroni/igad104.1963

**Published:** 2023-12-21

**Authors:** Samaneh Farsijani, Jane Cauley, Peggy Cawthon, Lisa Langsetmo, Eric Orwoll, Anne Newman, Deborah Kado

**Affiliations:** University of Pittsburgh, Pittsburgh, Pennsylvania, United States; University of Pittsburgh, Pittsburgh, Pennsylvania, United States; California Pacific Medical Center, Research Institute, San Francisco, California, United States; Minneapolis VA Health Care System, Minneapolis, Minnesota, United States; OHSU, Portland, Oregon, United States; University, Pittsburgh, Pennsylvania, United States; Stanford, Stanford, California, United States

## Abstract

While gut dysbiosis has been linked to frailty in aging, its association with early mobility impairments is unclear. Here, our primary goal was to determine the cross-sectional associations between walking speed and gut microbiome in 740 older men (84±4y) from MrOS with available stool samples and 400m walking speed measured in 2014–16. We also analyzed the retrospective longitudinal associations between changes in 6-meter walking speed (from 2005-06 to 2014-16) and gut microbiome composition among participants with available data (702/740). The gut microbiome composition was determined by 16S sequencing (DADA2 and SILVA). We examined diversity, taxa abundance (by ANCOM-BC), and performed network analysis (by NetCoMi) to uncover microbial communities interactions by walking speed levels. Higher walking speed (m/s) was associated with greater microbiome Shannon α-diversity (R=0.11; P=0.004). Decline in walking speed was associated with lower Shannon α-diversity (R=0.07; P=0.054). Faster walking speed and less decline in walking speed were associated with higher abundance of genus-level bacteria that produce short-chain fatty acids, and possess anti-inflammatory properties, including Paraprevotella, Fusicatenibacter, and Alistipes, adjusting for age, race, site, education, health, marital status, weight, height, physical activity, batch, medications, energy, and fiber intake (P< 0.05). The gut microbiome networks of participants in the first vs. last quartile of walking speed (≤0.9 vs. ≥1.2 m/s) exhibited distinct characteristics, including different cluster numbers, hubs, and centrality measures (P< 0.05). Faster walking speed and its less decline were associated with higher gut microbiome diversity, suggesting potential role of microbiome in preserving mobility in aging.

